# A systematic review to assess the use of psilocybin in the treatment of headaches

**DOI:** 10.1192/j.eurpsy.2023.1286

**Published:** 2023-07-19

**Authors:** S. Bhanot, M. C. Q. Lin, S. Bains, A. Monroe, V. W. L. Tsang

**Affiliations:** 1 Biochemistry and Biomedical Sciences, McMaster University, Hamilton; 2 University of British Columbia, Vancouver; 3 McMaster University, Hamilton; 4 Department of Psychiatry, University of British Columbia, Vancouver, Canada

## Abstract

**Introduction:**

Psilocybin is a naturally occurring psychedelic compound whose effects have been seen in studies for treatment of depression, anxiety and pain management. Given its structural similarities to 5-hydroxytryptamine, a monoamine controlling brain modulation of pain input, preliminary studies sought to test serotonergic interactions of psilocybin with headaches.

**Objectives:**

Explore efficacy of psilocybin as treatment for individuals with headaches, including migraines, essential headaches, cluster headaches and unclassified head pains.

**Methods:**

Studies were found from six major databases, with inclusion criteria consisting of participants with any type of headache using psilocybin as a treatment. Each study was independently screened by two reviewers at two stages, with inconsistencies reviewed by a third, senior reviewer.

**Results:**

The systematic review evaluated eight articles. Benefits of macrodosing were explored in one study which reported higher levels of pain relief in comparison to microdosing and conventional pain medications. Top benefits of microdosing as reported by participants included convenience, perceived safety and reduced side effects when compared to hallucinogenic doses of psilocybin. Participants across five studies reported improvements to their headaches as characterized by changes in frequency, intensity, duration and remission period. Reported improvements were clinically significant in the six studies and statistically significant in three papers. With psilocybin intervention, two studies reported a decrease in headache attack frequency, three studies reported a decrease in intensity, and one study indicated a decrease in duration. The greatest benefit reported was for psilocybin taken during a remission period, with the average length of that remission period between headaches extending for 91% of participants. One study focused on the dosages of psilocybin in relation to its efficacy, indicating that there was more headache pain relief amongst macrodosers, with a difference of 12.3% of participants experiencing pain reduction 3 days after dosage in comparison to microdosers. 18% of participants who experienced essential headaches also experienced hallucinations as a result of ingested psilocybin. Others showed a temporary increase in symptoms of anxiety and pain - 5.3% with microdosing and 14.1% macrodosing. One study observed an increase in average arterial pressure after ingestion.

**Conclusions:**

Six of eight screened papers showed that psilocybin was clinically significant in the treatment of headaches as captured through self-reports. While the first controlled study for psilocybin use for headaches was detailed in this study, psilocybin remains illegal in many countries, presenting a need for further regulated research.

**Disclosure of Interest:**

None Declared

